# Structural Insights into *Clostridium perfringens* Delta Toxin Pore Formation

**DOI:** 10.1371/journal.pone.0066673

**Published:** 2013-06-21

**Authors:** Jessica Huyet, Claire E. Naylor, Christos G. Savva, Maryse Gibert, Michel R. Popoff, Ajit K. Basak

**Affiliations:** 1 Department of Biological Sciences, Birkbeck College, London, United Kingdom; 2 Department of Microbiology, Pasteur Institute, Paris, France; National Research Council of Italy, Italy

## Abstract

*Clostridium perfringens* Delta toxin is one of the three hemolysin-like proteins produced by *C. perfringens* type C and possibly type B strains. One of the others, NetB, has been shown to be the major cause of Avian Nectrotic Enteritis, which following the reduction in use of antibiotics as growth promoters, has become an emerging disease of industrial poultry. Delta toxin itself is cytotoxic to the wide range of human and animal macrophages and platelets that present G_M2_ ganglioside on their membranes. It has sequence similarity with *Staphylococcus aureus* β-pore forming toxins and is expected to heptamerize and form pores in the lipid bilayer of host cell membranes. Nevertheless, its exact mode of action remains undetermined. Here we report the 2.4 Å crystal structure of monomeric Delta toxin. The superposition of this structure with the structure of the phospholipid-bound F component of *S. aureus* leucocidin (LukF) revealed that the glycerol molecules bound to Delta toxin and the phospholipids in LukF are accommodated in the same hydrophobic clefts, corresponding to where the toxin is expected to latch onto the membrane, though the binding sites show significant differences. From structure-based sequence alignment with the known structure of staphylococcal α-hemolysin, a model of the Delta toxin pore form has been built. Using electron microscopy, we have validated our model and characterized the Delta toxin pore on liposomes. These results highlight both similarities and differences in the mechanism of Delta toxin (and by extension NetB) cytotoxicity from that of the staphylococcal pore-forming toxins.

## Introduction

Delta toxin is one of three toxins expressed by *C. perfringens* that have approximately 25% sequence identity with the leucocidin family of pore-forming toxins secreted by *S. aureus* (supplementary Fig. 1) [1], [2]. All three of the toxins have been shown experimentally to assemble into oligomeric pores on cell surfaces [1]. Delta toxin has been found to be hemolytic to red blood cells from even-toed ungulates and cytotoxic to a wide range of white blood cells, such as macrophages, monocytes and blood platelets of humans and various animals [3]–[6]. The other two *C. perfringens* toxins are necrotic enteritis toxin B (NetB), the recently discovered cause of Avian Necrotic Enteritis [7] and Beta toxin, the cause of Pig Bel in humans and necrotic enteritis in animals including pigs, goats and sheep [8]. Antibiotics have been added to animal feedstuffs to promote growth for some time [9]. Following concern about increasing microbial resistance, some governments are requiring reductions of these additives [10]. As a consequence there are renewed concerns about the emergence of infectious diseases in industrial farming. Although related to the staphylococcal pore-forming toxins, Delta toxin, NetB and Beta toxin form a separate subgroup, and likely a number of unique features. These three toxins are of particular interest as NetB and Beta toxin have both been shown to have significant links to animal disease and Delta toxin is known to be cytotoxic, and their similarities mean information on one is likely to be applicable to the others.

**Figure 1:**
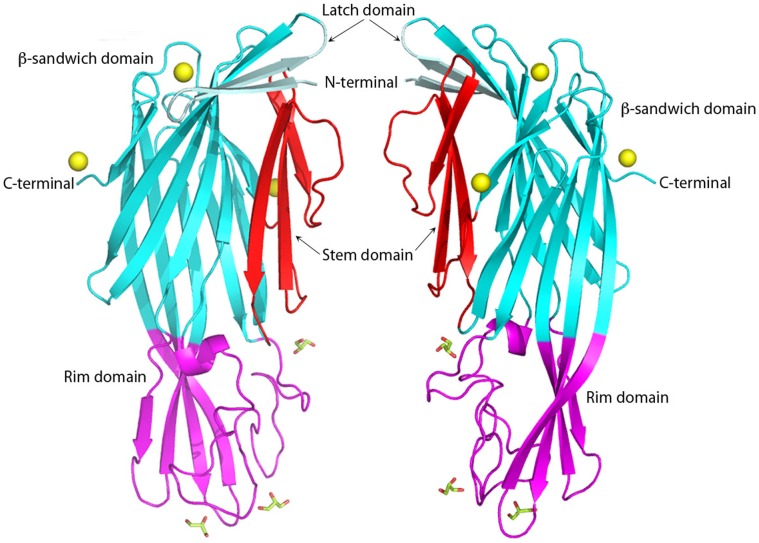
Two views at 180°C of the *C.* *perfringens* Delta toxin structure in cartoon representation. Latch domain, β-sandwich domain, Stem domain and rim domain are colored in pale cyan, cyan, red and magenta, respectively. Glycerol molecules are shown as sticks. Zinc molecules are depicted as yellow balls. Figures 1–4 are produced with PyMol.

Delta toxin’s selectivity for white blood cells and human and animal platelets has been linked to its specific binding to gangliosides, particularly to the monosialic ganglioside 2 (G_M2_) [6]. It also binds G_M2_ isolated from cell membranes and either used to form liposomes or immobilized on polystyrene beads [1], [11]. Gangliosides present on cell surfaces, participate in membrane organization and can act as receptors [12]. Therefore it seems likely that G_M2_ acts as the receptor for this toxin, though a role for a membrane protein receptor has not been completely ruled out [1].

The leucocidin family of bacterial toxins are initially secreted as water-soluble monomers, which recognize receptors on the surface of cells and bind to the cellular membranes as monomers. Subsequently, they oligomerize on the cell surface to form prepores that insert amphipathic hairpins into the lipid bilayer to form an oligomeric β-barrel pore [13]. The *S. aureus* members of this family include α-hemolysin (αHL), γ-hemolysin (γHL), leucocidin (Luk) and Panton-Valentine leukocidin (PV-Luk) [14]. αHL forms a homoheptamer [15], while γHL, Luk and PV-Luk are bicomponent toxins where an F component protein binds the membrane initially, followed by an S component protein to form a heterodimer [16] and the heterodimers then oligomerise to form the pore [17], [18]. In γHL, Luk and PV-Luk, the F and S components are LukF and Hlg2, LukF and LukS and LukF-PV and LukS-PV, respectively. Sequence alignment (supplementary Fig. 1) reveals that the LukF proteins are around 70% identical to each other, as are LukS proteins to each other, however, LukF and LukS share only approximately 30% sequence identity between them. αHL shares more sequence identity with LukF proteins (∼30%) than LukS proteins (∼20%). All three *C. perfringens* toxins are active as homo-oligomers, and have more similarity to αHL and the LukF proteins (∼27%) than LukS proteins (∼21%).

There are structures available for a number of the *S. aureus* proteins. These include the water-soluble monomeric forms of LukF [19], LukF-PV [20] and LukS-PV [21], the homoheptameric pore form of αHL [15] and the hetero-octomeric pore-form of γHL [22]. The structures are all similar and are organized in four domains: an N-terminal latch domain, a β-sandwich domain, then a central domain called the stem domain and finally a C-terminal region rich in β-strands, called the rim domain. The main difference between the monomeric and oligomeric state is the folding and position of the stem domain. In the monomeric form, it is folded into β-strands, which are packed against the N-terminal β-sandwich domain [19], [20], whereas in the oligomeric form it unravels to form a transmembrane β-hairpin. [15], [22]–[24]. After oligomerization, the β-hairpin from each monomer assembles to form an anti-parallel β-barrel that inserts into the membrane [15], [22]–[24]. In αHL, the latch domain is also extended to form stabilising interactions with the adjacent monomer in the heptamer [15], [23], in γHL, however, this domain is disordered [22], and it is not required for cytotoxic activity [25]. Very recently, the structures of the heptameric pore [24] and soluble monomer [26] of NetB have become available confirming the overall similarity of this subgroup to the staphylococcal toxins, though there are a number of distinct features in the receptor binding domain.

Here, we present the monomeric structure of Delta toxin at 2.4 Å resolution, determined by X-ray crystallography. The structure revealed a fold that is similar to NetB and the staphylococcal β-pore forming toxins. From structure-based sequence alignment and superposition of the structures of Delta toxin, we show that Delta toxin shares many characteristics of the LukF family members and that this has implications for membrane binding. However, we were able to identify differences that are likely to be linked to difference in cell specificity between the groups of toxins. In addition, we built a 3D model of the heptameric pore form of Delta toxin based on the staphylococcal α-hemolysin heptamer. By using electron microscopy we validated our model and further characterized the pore form of Delta toxin.

## Materials and Methods

### Protein Expression, Purification and Crystallization

*C. perfringens* Delta toxin was over-expressed, purified and crystallized as reported previously [1], [27]. Briefly, Delta toxin was expressed as a N-terminal His-tagged protein in *E. Coli* BL21, purified using affinity chromatography and subsequently concentrated to 6.6 mg/ml in 20 mM Tris-HCl pH 8.0, 100 mM imidazole, 50 mM NaCl and 5% glycerol. X-ray diffraction quality crystals grew in the presence of 100 mM MES-NaOH pH 6.0, 25–30% polyethylene glycol monomethyl ether 550, 25 mM ZnSO_4_. The crystals belong to the orthorhombic space group P2_1_2_1_2 with cell dimensions a = 112.9, b = 49.7 and c = 58.5 Å. Calculations based on Matthews coefficient indicated that there is one monomer in the asymmetric unit with a V_M_ of 2.58 Å^3^.Da^−1^ corresponding to a solvent content of 52.4%.

### Data Collection, Processing and Phasing

The data were collected on beamline ID29 at ESRF in Grenoble, France. The diffraction data were processed with MOSFLM [28] and scaled and merged using SCALA [29]. The CCP4 suite of programs [30] was used for all subsequent steps. Molecular replacement was carried out successfully with a number of different hemolysin-like structures. The best results used the S component of *S. aureus* Panton-Valentine leucocidin (PDB ID 1T5R [21], as the search model and the program Phaser [31]. This gave a Z-score of 11.6 and a log-likelihood gain of 123.4 following placement of a single copy in the asymmetric unit, and an R-factor and R-free of 41.8 and 44.9% respectively following an initial rigid-body refinement round.

### Model Building and Refinement

Alternating rounds of refinement in Phenix [32] and manual rebuilding in Coot [33] were carried out until no further improvement in R-factor and R-free could be achieved. The final model comprises residues 9 to 290, three Zn ions, three glycerol molecules, one imidazole and 139 water molecules. The final crystallographic R-factor and R-free are 17.9% and 22.8% respectively, and the model has good geometry as assessed by Molprobity [34].

### Molecular Modeling

We choose to model a Delta toxin heptamer based on the structure of αHL [15], [23] for two reasons. Firstly, we excluded a model based on γHL [22] because Delta toxin forms homo-oligomers like αHL, rather than hetero-oligomers as seen in γHL. Secondly, αHL has detectable sequence homology to Delta toxin, whereas the more recently solved (and structurally related) *Vibrio cholerae* cytolysin heptamer [35] has inserted domains and much lower sequence homology. Delta toxin was sequence aligned with a number of other known hemolysin-like atomic structures using ClustalW [36] and αHL (PDB ID 7AHL) [15], and residues corresponding to the stem and latch domains in αHL were deleted from the final refined model of Delta toxin. The coordinates of the refined Delta toxin minus these two regions were then optimally superposed onto the A molecule from αHL using SSM [37] in CCP4. The residues corresponding to the latch and stem domains from 7AHL where mutated to their corresponding residues in Delta toxin using Chainsaw [38] from CCP4 and these two domains added to the Delta toxin. This model of a Delta toxin pore-form monomer was then superposed on each of the 7 monomers in 7AHL in turn to form a model of a heptameric Delta toxin pore. The model was then energy-minimised in Phenix [32].

### Oligomerization on Cells

Recombinant Delta toxin was produced in *E. coli* from pET28 vector and purified on cobalt column as previously described [1]. The His-tag was removed by thrombin (Novagen) and was labeled with Cy3 according to the manufacturer’s recommendations (GE Healthcare). Cy3-Delta toxin was checked for cytotoxic activity on HeLa cells.

HeLa cells were incubated with 5 µg/ml Cy3-Delta toxin for 30min at 4°C (lane 1), 5 µg/ml for 30 min at 37°C (lane 2), or 10 µg/ml for 30 min at 37°C (lane 3) in DMEM medium containing 0.1% BSA. Then the cells were washed three times with PBS and lysed with Tris-HCl 10 mM pH 7.5 containing Triton X100 1% and DNAse 50 µg/ml. The cell lysates were elctrophoresed in a SDS-containing 10% polyacrylamide gel and scanned for fluorescence with a Typhoon scanner using a wavelength of 532 nm.

### Electron Microscopy

Samples for electron microscopy were prepared as follows. A mixture of lipids (Egg phosphatidylcholine:Egg phosphatidylglycerol:cholesterol at a molar ratio of 4∶1:5, Avanti polar lipids) was dried under nitrogen and rehydrated in buffer (50 mM Tris pH 8.0, 150 mM NaCl). The rehydrated lipids were incubated at 37°C for 30 minutes followed by vigorous vortexing. The suspension was then subjected to two rounds of freezing in liquid nitrogen followed by thawing at 37°C. Finally, the lipid suspension was extruded through a 100 nm pore filter 21 times. Delta toxin was mixed with 150 µM lipid to give a final Delta toxin heptamer:liposome ratio of 50∶1 and incubated at 37°C. A 5 µl sample was then applied to formvar-carbon coated grid that had been freshly glow-discharged. Specimens were observed on a Phillips T12 transmission electron microscope operating at 120 kV.

## Results

### Structure Determination of the X-ray Crystal Structure

In an effort to determine the structure of *C. perfringens* pore-forming toxins, we cloned, over-expressed, purified, and crystallized Delta toxin [1], [27], resulting in crystals that diffracted up to 2.4 Å resolution. Analysis of the diffraction pattern and systematic absences led to Delta toxin crystals being assigned to the orthorhombic space group P2_1_2_1_2, with unit-cell dimensions a = 112.93, b = 49.66, c = 58.48 Å. The Matthews coefficient (V_M_ of 2.58 Å^3^.Da^−1^) suggested that the asymmetric unit contained one molecule of Delta toxin, corresponding to a crystal solvent content of 52.4%.

*C. perfringens* Delta toxin structure was determined by molecular replacement using Phaser [31] and LukS-PV (PDB ID: 1T5R; [21]) as the model and yielded excellent quality electron density maps. The model was refined using Phenix [32] and built with Coot [33]. It includes a monomer of Delta toxin, comprising 282 residues (residues Ile9 to Ser290), 139 water molecules, three glycerol molecules, one imidazole and three zinc molecules. The final values of R-factor and R-free are 17.9% and 22.8%, respectively (Table 1). The final refined co-ordinates were submitted to the Protein DataBank with PDB ID: 2YGT.

**Table 1:** Data collection and structure refinement statistics.

Synchrotron/beamline	ESRF ID29
**Crystal parameters**	
Space group	P2_1_2_1_2
Cell dimensions (Å)	a = 112.93, b = 49.66, c = 58.48
Angles (û)	α = β = γ = 90°
**Data collection**	
Wavelength (Å)	1.0332
Resolution limit (Å)	100.0–2.40 (2.53–2.40)
Mosaicity	0.29û
R_merge_	0.15 (0.69)
Total number of observations	140,319 (20,111)
Total number unique	13,409 (1898)
Mean I/σI	15.8 (3.5)
Completeness (%)	99.9 (99.8)
Multiplicity	10.4 (10.6)
**Refinement**	
Protein atoms in model	2173
Solvent atoms in model	250
R_working_	0.179
R_free_	0.228^a^
**RMSD from ideal geometry** b	
Bond lengths (Å)	0.08
Bond angles (°)	0.772
Wilson B-factor (Å^2^)	38.1
Mean B-factor of protein atoms (Å^2^)	29.6
**Ramachandran plot**	
Most favoured (%)	94.7
Outlier (%)	0.0
PDB ID code	2YGT

R_merge_ = Σ|I_i_ – bI_i_N|/ΣI_i._

R_working_ = Σ|F_o_ – F_c_|/ΣF_o_.

R_free_ is the R-factor calculated for the cross-validated test set of reflections.

a R_free_ is 4.9% of reflections.

b As defined by MOLPROBITY.

### Monomeric form of Delta Toxin Determined by X-ray Crystallography

*C. perfringens* Delta toxin structure has an elongated ellipsoid shape composed of eighteen β-strands and three short helical fragments: two 3_10_ helices and one α-helix (Fig. 1). These secondary structure elements are arranged into three structural domains: a β-sandwich domain, a stem domain and a rim domain (Fig. 1). The β-sandwich domain is made up of a sandwich of two anti-parallel β-sheets, composed of six and seven β-strands, respectively. Three β-strands are folded into an anti-parallel β-sheet to form the stem domain. The rim domain consists of one α-helix turn and an anti-parallel β-sheet of four β-strands (Fig. 1).

### Comparison of Delta Toxin with Related Proteins

**Figure 2:**
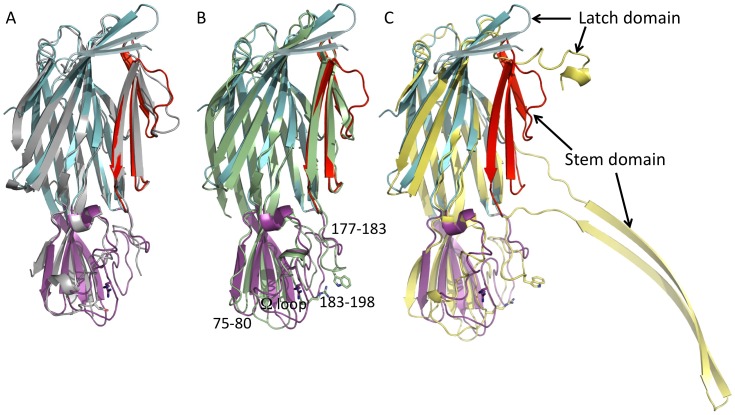
Superposition of *C.* *perfringens* Delta toxin structure with γHL-Hlg2 in grey (PDB ID: 3B07) (A), with γHL-LukF in pale green (PDB ID: 3B07) (B) and with a monomer of the αHL of *S. aureus* in yellow (PDB ID: 7AHL) (C). Colors for the different domains of *C. perfringens* Delta toxin have been kept as for Fig. 1. The conserved Arginine and Tryptophan associated with phospholipid binding are shown as sticks. Loops in the rim domain that differ in the various toxins are identified in (B) by their residue numbers in Delta.

To compare Delta toxin with the staphylococcal toxins, we superposed their structures and performed a maximum-likelihood structure-based sequence alignment using Theseus-3D [39], [40]. The superposition together with the multiple sequence alignment shows that, despite a relatively low sequence identity between Delta toxin and the staphylococcal β-pore forming toxins (less than 30%), the secondary structure elements are conserved across the whole group (Fig. S1 and Fig. 2). The root mean square deviation for all Cα-atoms that have equivalents in all aligned structures, excluding the stem domain (198 atoms or 70% of the Cα-atoms in Delta toxin), is between 0.64 and 2.34 Å (Table 2).

**Table 2:** Root-mean-square deviation in Angstroms, between the 198 Cα-atoms that have equivalents in all structures, excluding the stem domain, following Maximum-Likelihood-based multiple sequence alignment with Theseus-3D [39], [40].

RMSD (Å)	Delta	αHL(7ahl)	Hlg2-γHL(3b07)	LukF-γHL(3b07)	LukS-PV(1t5r)	LukF-PV(1pvl)
LukF (1lkf)	1.82	1.67	0.75	2.34	2.25	0.64
LukF-PV (1pvl)	1.67	1.61	0.87	2.22	2.14	
LukS-PV (1t5r)	1.80	1.78	2.19	1.20		
LukF-γHL(3b07)	1.76	1.62	2.25			
Hlg2-γHL(3b07)	1.80	1.77				
αHL(7ahl)	1.74					

Delta toxin shows a novel conformation of the N-terminal latch domain (Fig. 2). In αHL, the N-terminal residues are extended and form interactions with the adjacent monomer in the heptamer [15]. In both components of the γHL pore [22], the recently solved structure of the NetB pore form and all monomeric S component structures [21] these residues are disordered. In the monomeric NetB [26] structure, this latch conformation is partially, but not completely, ordered, in a similar manner to Delta toxin. However, in monomeric F component structures [19], [20] they extend along the whole length of the β-sandwich domain to form an additional strand. In Delta toxin, the N-terminal residues form a β-hairpin that extends halfway along the β-sandwich domain and creates a cap above the folded, monomeric stem domain residues (Fig. 1 and Fig. 2).

As has been well noted previously, the main difference between both Delta toxin and the monomeric leukocidins and the heptameric pore structures (αHL, γHL and NetB) is that the stem domain adopts an extended β-hairpin conformation in the mature pore structures [15], [22]–[24], while it is folded back towards the β-sandwich domain in the water-soluble form structures [19]–[21].

As described earlier, a number of the staphylococcal toxins are bicomponent, with both F and S components required for functionality. Other authors have shown that the F and S components differ most in their rim domains [21]. By comparing Delta toxin to the F and S components, we observed that the rim domain is more similar to the F component than the S (Fig. 2A and B). In Delta toxin, residues 75–80 form a short turn similar to that for component F rather than the extended loop seen in S component structures (Fig. 2B). Delta toxin residues 183–193 form an extended loop present in component F but not in component S (Fig. 2B). The Ω loop formed by residues 199–209 of Delta toxin follows the conformation seen in component F structures; in S component structures this loop is flipped. Finally, residues 258–261 in Delta toxin form a loop at the base of the rim domain, similar to that seen in component F, while in component S this loop is longer due to the insertion of 5 amino acids. There are differences, however between Delta toxin and F component structures. Though we have already commented that residues 75–80 form an F-type like short turn, rather than an S-type extended loop, the conformation of this turn is quite different from the F component. In addition, the Ω loop in Delta toxin is shorter than that seen in any of the *S. aureus* proteins. Finally, Delta toxin has a four-residue deletion in the residue 177–183 loop (Fig. 2B) relative to the *S. aureus* proteins. This deletion means that Delta toxin does not possess a Tryptophan (residue 177 in LukF; Fig. 2B) that is important for phosphatidylcholine binding in the *S. aureus* proteins [41], [42]. A similar conformation to that of Delta toxin of the rim domain is seen in both NetB structures [24], [26].

### Model of the Heptameric Pore Form of Delta Toxin

**Figure 3:**
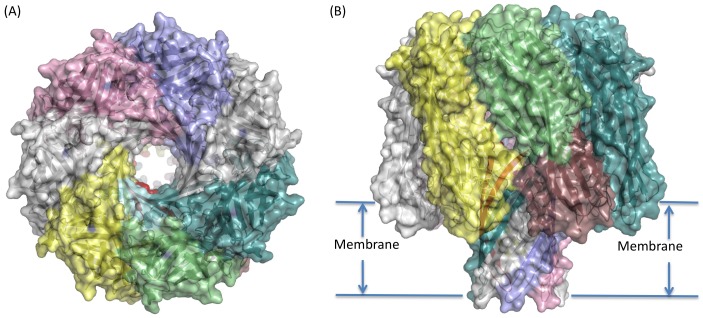
Model of the Delta toxin heptameric pore shown in cartoon representation with a semi-transparent surface. Chain A is coloured cyan for the latch domain, pale green for the β-sandwich, red for the stem and raspberry for the stem domains. Remaining chains shown in single colour (pale teal, grey, lilac, pink white and yellow). (A) Top (looking down at extra-cellular face) and (B) side-view, indicating possible membrane location.

The Delta toxin heptameric pore model was generated by splicing the β-hairpin and latch domains of αHL [15], [23] to the rim and β-sandwich domains of Delta toxin (Fig. 3). It has no clashes and is stable in energy minimisation. The heptamer is similar to the Delta toxin oligomer seen by electron microscopy (see below) and has an unobscured central channel for ion conduction (Fig. 3). In addition, the recently solved structure of the NetB oligomer [24], to which Delta toxin is closely related (approximately 43% sequence identity), is also heptameric and closely resembles our model, with an Cα-atom RMSD of 1.2 Å over 261 residues, including the stem domain, between a monomer from the Delta heptameric model and one from the NetB heptamer, and 1.7 Å over 1827 matched Cα in the heptamers. Interestingly, the buried surface area in this model, at approximately 2300 Å^2^ per interface, though still extensive, is significantly smaller than that seen in αHL (approximately 2800 Å^2^ per interface), and all but one of the salt bridges present in αHL have been lost in this model. The reduction in contact regions between monomers in the Delta toxin can be seen in holes in the side of the pore in the β-sandwich domain (Fig. 3).

Both the recently published NetB heptamer [24] and γHL octamer [22] also appear to show a similarly reduced monomer interface (both with around 2200 Å^2^ buried per interface). However, in both these complexes the latch domain is disordered and not contributing to the interface. We have modelled a latch domain into the Delta heptamer, and in both our model and αHL, the latch domain contributes approximately 640 Å^2^ buried surface area and a number of hydrogen bonds to the interface. This suggests that the main heptamer interface, including the stem domain is similar in size in αHL, NetB and γHL, but significantly smaller in our Delta toxin model.

### Implications for Membrane Binding in Delta Toxin

**Figure 4:**
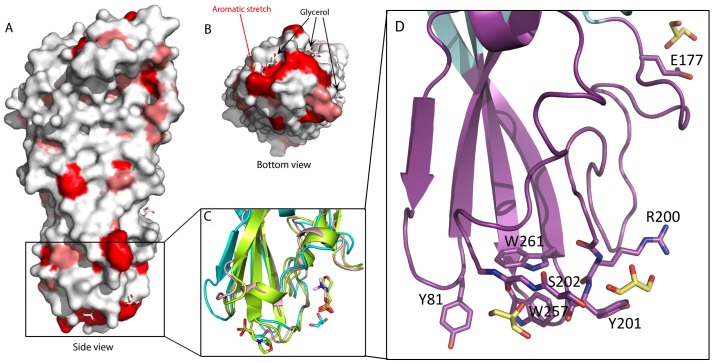
*C.* *perfringens* Delta toxin structure surface from the side (A) and from the bottom of the rim domain (B). Aliphatic residues (Ala, Val, Ile, Leu, Met) are in salmon and aromatic residues (Phe, Trp, Typ) are in red. Glycerol molecules are shown as grey sticks. In C, a cartoon representation of the glycerol molecules binding region of *C. perfringens* Delta toxin in cyan superposed with *S. aureus* LukF in lemon (PDB ID: 3LKF; [19]) and LukF-PV in pink (PDB ID: 1PVL; [20]). In D, the glycerol molecule binding region of Delta toxin, coloured as for Fig. 1 showing the residues involved in binding glycerol which are shown as lemon sticks.

A prerequisite of Delta toxin action is binding to the target membrane. With the aim of defining which region is involved in this interaction, we studied the nature of the solvent-exposed residues in Delta toxin. When studying the hydrophobicity of the solvent-exposed residues, we found that a large number of hydrophobic residues are located in the rim domain. In particular, there is an aromatic patch composed of seven residues (Tyr81, Tyr182, Trp187, Tyr191, Tyr201, Trp257, Trp261, Tyr266) at the base of this domain (Fig. 4A and B). These residues are partly composed of and close to the Ω and shortened residue 75–80 loops described previously. The presence of hydrophobic, and more specifically, aromatic residues at this particular location has already been highlighted for the staphylococcal leucocidins [19], [20] and has been linked to their interaction with phospholipid head groups. However, with the exception of Tyr 191, the exact residue positions are not in general conserved between Delta toxin and the leukocidins, suggesting a general similarity in membrane recognition, while the details of the mechanism are different.

### Small Molecules Bound to Delta Toxin

There are three glycerol molecules bound to the hydrophobic region of the rim domain (Fig. 4). Two (named Gol5 and Gol6 in the rest of this manuscript) are bound to opposite sides of the 199–209 Ω loop, close to the patch of hydrophobic residues. Gol5 is bound to a pocket formed by Tyr81 and Trp257 and Trp261 and forms hydrogen bonds with the mainchain of residues 201–203. Gol6 packs against Tyr201 and Arg200 and forms hydrogen bonds with residues 200–202. The third glycerol, referred to as Gol7 here, is bound at the top of the rim domain, close to the base of the folded stem domain, it is hydrogen bonded to the sidechain of Glu177. There are no small molecules observed bound to the NetB heptamer [24], however there is an ethylene glycol bound to monomeric NetB at an equivalent location to Gol5 [26].

The *S. aureus* F components and αHL have been cocrystallised with a number different small molecules. LukF was cocrystallized with dipropanoyl phosphatidyl choline, and the phosphocholine headgroup was seen bound in a pocket formed by Trp177 and Arg198 [19]. Interestingly, when Delta toxin and LukF are superposed, the glycerol of Delta toxin is positioned where the glycerol moiety of the phosphatidylcholine would be expected to be in the LukF structure, if it had been ordered (Fig. 4C). In addition, MES-NaOH molecules were found in the LukF-PV structure, and were proposed to be mimicking lipid head groups, (no MES-NaOH was used in crystallisation; 20,21) these MES molecules were also bound in the Trp177/Arg198 pocket. When the proteins are superposed, Gol5 and Gol6 in Delta toxin overlap with these MES-NaOH molecules. Finally, both the LukF in γHL [22] and a recent determination of αHL heptamer structure [23] have 2-methyl-2,4-pentanediol (MPD) bound in the Trp177/Arg198 pocket, overlapping with Gol6 in Delta toxin when the proteins are superposed.

One imidazole molecule is bound between the sidechains of Asp34 and Arg287 at a crystallographic interface. Imidazole is present at significant concentration in the crystallisation conditions, is bound to non-conserved residues, and is also in a non-physiological environment created by the presence of the non-biological crystallographic interface. It is unlikely this molecule has any biological significance. There are also three bound zinc molecules. Zinc was also present at significant quantity in the crystallisation conditions, and all three zinc ions are bound either to charged residues at the protein surface, which with one exception, are not conserved, or, in one case, bound imidazole. The zinc is therefore unlikely to be biologically significant.

### Characterization of Delta Toxin Pores by Electron Microscopy and Fluorescence Imaging

**Figure 5:**
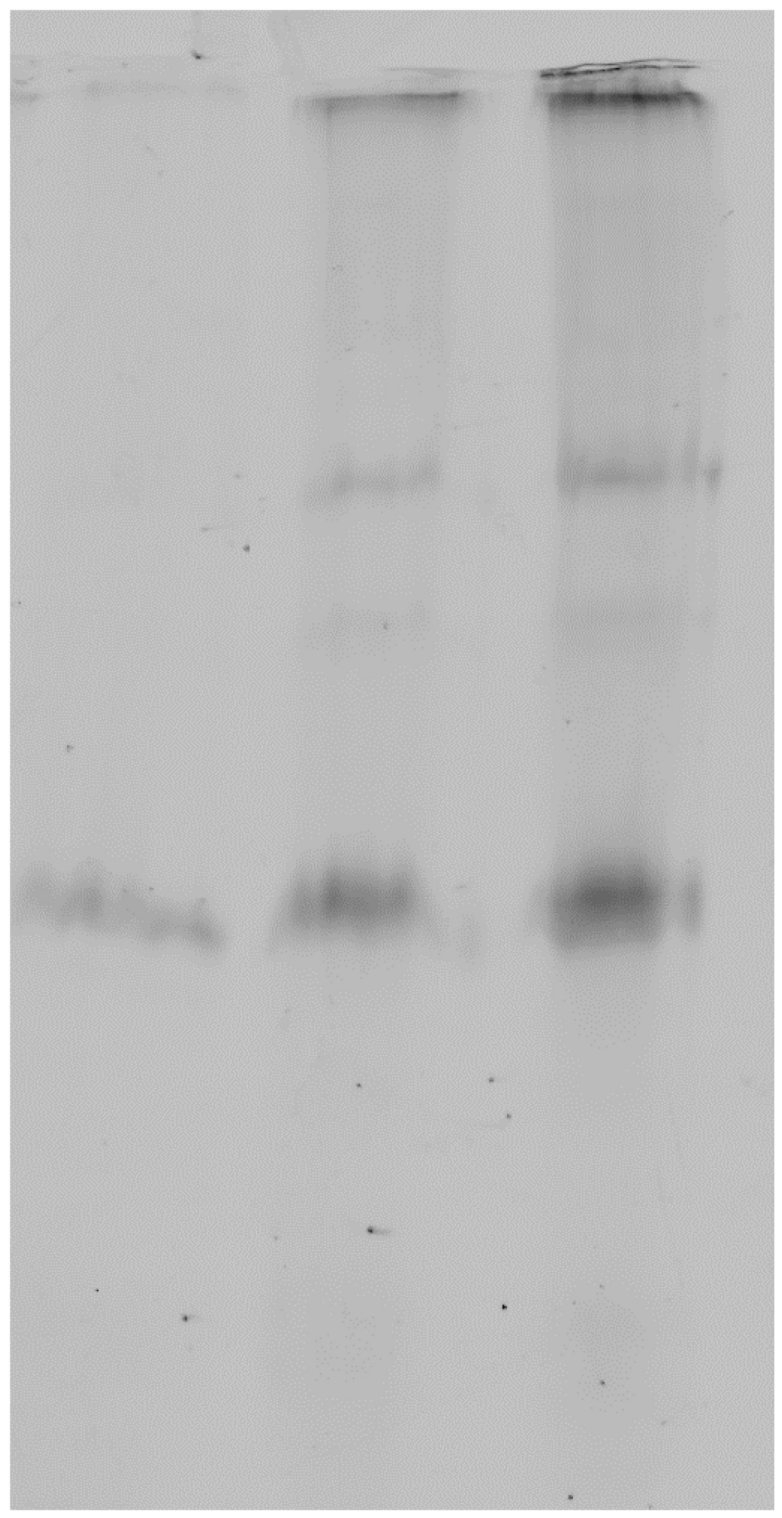
HeLa cells were incubated with 5 µg/ml Cy3-Delta toxin for 30min at 4°C (lane 1), 5 µg/ml for 30 min at 37°C (lane 2), or 10 µg/ml for 30 min at 37°C (lane 3) in DMEM medium containing 0.1% BSA. After washing, the cell lysates were elctrophoresed in a SDS-containing 10% polyacrylamide gel without reducing agent and scanned for fluorescence.

**Figure 6:**
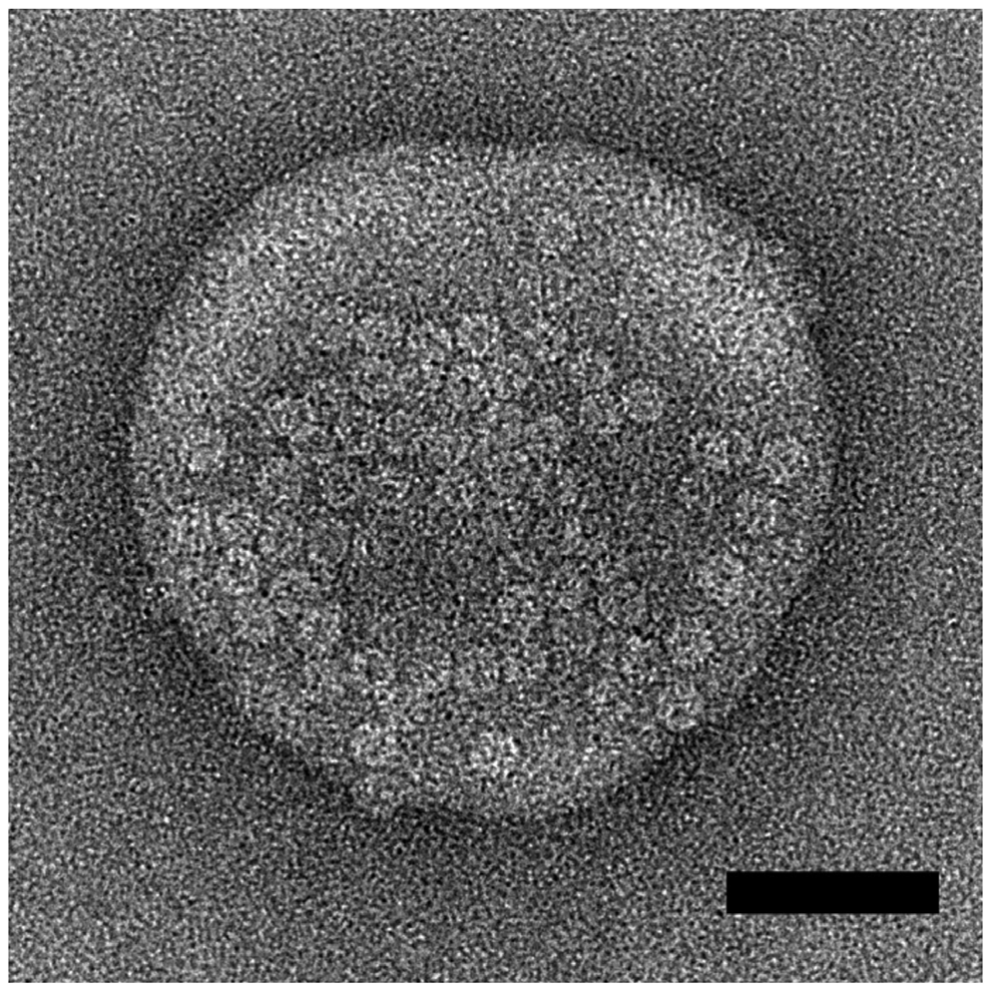
Electron micrographs of liposomes incubated with Delta toxin. Scale bars correspond to 50 nm.

In order to confirm that Delta toxin is able to bind membranes and oligomerize in a manner similar to other staphylococcal β-pore forming toxins, we observed Delta toxin on the surface of sensitive cells by fluorescence imaging and in the presence of liposomes by electron microscopy. HeLa cells were incubated with fluorescent Delta toxin and oligomers were visualized by SDS-PAGE and fluorescence imaging. Delta toxin formed oligomers only when incubated at 37°C with cells and not at 4°C (Fig. 5). Electron microscopic observation of the toxin-liposome mixtures revealed liposomes heavily packed with toxin oligomers (Fig. 6). The oligomers are ring-shaped and measure approximately 10 nm in diameter. This dimension closely matches the reported diameter of the αHL pore form at its maximum dimension. These observations confirm that Delta toxin is able to bind and oligomerize on lipid bilayers.

## Discussion

Antibiotics have been added to animal feedstuffs to promote growth for some time [9]. Following concern about increasing microbial resistance, some governements are requiring reductions of these additives [10]. As a consequence there are renewed concerns about the emergence of infectious diseases in industrial farming. The leukocidin-like toxins secreted by *C. perfringens* are of interest in connection with this, as NetB and Beta toxin have been shown to have significant links with these diseases, while Delta toxin is cytotoxic, as discussed earlier [1], [7], [8]. In this study, we have described the monomeric structure of *C. perfringens* Delta toxin and a model of its oligomeric form that is supported by electron microscopy views of liposomes surrounded by pores. As expected, the monomeric structure has a fold that is homologous to those of the previously solved structures of the monomeric *S.* *aureus* leukocidin structures, however it suggests differences in membrane recognition and headgroup specificity which are likely to be of importance in the design of therapeutics or novel vaccines for the animal disease caused by the *C. perfringens* group of pore-forming toxins.

In Delta toxin we have observed bound glycerol in a location similar to that in which lipid and amphipathic moleules have been observed binding both to αHL and LukF [19], [23]. Ethylene glycol has also been observed binding to this location in NetB [26]. In the bicomponent pores, F component proteins have been associated with initial phosphatidylcholine binding prior to binding of LukS and its recognition by any specific receptors [42]. Reflecting this difference in function, the rim domain, which interacts most closely with the membrane surface in the oligomeric structures, is the location of the largest structural differences between F and S components [21]. The site of lipid and amphipathic molecule binding in LukF is eliminated in LukS by loss of the conserved residues associated with membrane binding, the change in Ω-loop conformation and by the insertion of long loops that occupy the space normally taken by the ligand. It is noticeable that in all three structures of homo-heptameric pore forming toxins solved to date (αHL, NetB and Delta toxin), these toxins are more similar to LukF than LukS. In addition, both αHL and now Delta toxin have small molecules bound to the lipid binding site identified in LukF [19], [20], [23]. Membrane-binding is an essential precursor to insertion of the pore into the lipid bilayer, regardless of any particular specificity and thus the homo-oligomers have retained the LukF lipid binding site at the cost of any specificity conferred by the LukS rim domain.

Nevertheless, Delta toxin still requires specificity as it has been shown to be cytotoxic only to cells expressing G_M2_ in their membrane [3]–[5], [11]. There are aspects of the Delta toxin that might shed light on this specificity. The Delta toxin structure has three bound glycerol molecules, all located in the rim domain, which is mainly composed of hydrophobic and aromatic residues. As we have already described, two of these glycerol molecules are close to the Ω loop and a stretch of hydrophobic residues. Interestingly, the superposition of αHL and component F structures with bound small molecules [19], [20], [22], [23] and the Delta toxin structure revealed that hydrophobic molecules such as phosphatidylcholine, MPD or even MES-NaOH are found at the same location, close to the Ω loop, as two of the glycerol molecules. One of these two glycerol molecules interacts with Trp257, and replacement of the equivalent residue in NetB reduces cytotoxicity [24], [26]. The Ω loop contains an arginine that is conserved in all the pore-forming toxins except the S components, corresponding to Arg200 in Delta toxin, the other glycerol is interacting with this arginine, which has been shown to play a crucial role in these toxins. In *S. aureus* αHL, the mutation of the corresponding arginine (Arg200) to cysteine abolishes the ability of the toxin to bind its target cells, inducing a complete loss of hemolytic activity [43]. Similarly, the replacement of the corresponding arginine (Arg212) by a glutamate in *C. perfringens* Beta toxin impairs the toxin’s oligomerization and the binding to cell surface, resulting in a decrease of toxin lethal activity [44] and the replacement of the equivalent Arginine in NetB eliminates cytotoxicity [24], [26]. Likewise, it has been shown that the mutation of the corresponding arginine (Arg198) to threonine in *S. aureus* LukF reduces the hemolytic activity by impairing the membrane-binding and the hetero-oligomerization with Hlg2 and therefore the formation of the γHL pore [42]. One of the glycerol molecules in the Delta toxin structure is interacting with the Arg200 residue, and, it is likely that, in analogy with these related proteins, Arg200 in Delta toxin is important for cell surface recognition.

Interestingly, however, in αHL and component F, the recognised phospholipid binding site is completed by a conserved Tryptophan (Trp177 in LukF) [42]. We have already noted that Delta toxin has differences from the leukocidins. The most noticeable of these is the loss of the loop containing Trp177. It has been shown that G_M2_ ganglioside is the receptor for the Delta toxin, while the leukocidins bind phosphatidylcholine. The loss of the tryptophan containing loop enlarges the binding site as well as changing its chemical nature, and this may reflect the binding of G_M2_ ganglioside rather than phosphatidylcholine. It is interesting to note that all three *C. perfringens* toxins have lost the tryptophan containing loop (Fig. S1 and Fig. 2A), perhaps reflecting altered cell specificties for all these toxins compared to the staphylococcal proteins.

*C. perfringens* secretes a range of β-PFTs from a number of different families. Distinct from the hemolysin-like group of which Delta toxin is part, are *C. perfringens* enterotoxin and epsilon toxin. These toxins are structurally related to each other and are members of the Aerolysin family [45], [46] of β-PFTs, despite sharing no significant sequence homology with Aerolysin or one another. Aerolysin, from the bacterium *Aeromonas,* is the prototype of this toxin family which also encompasses animal and plant toxins, such as hydralysin and enterolobin, respectively [47]–[49]. *C.* *perfringens* enterotoxin and epsilon toxin are more elongated than Delta toxin, but like Delta toxin they contain three domains and form hexamers or heptamers. In these aerolysin-like toxins, the N-terminal domain is involved in binding to specific receptor, domain 2 contains an amphipatic β-hairpin forming the β-barrel, and the C-terminal domain is associated with oligomerization [45], [46]. Perfringolysin (PFO) also produced by *C. perfringens* is a representative of the cholesterol-dependent cytolysin (CDC) family. PFO retains a structural organization and mode of insertion into membrane similar to those of other β-PFTs. But PFO forms large pores resulting from the association of a large number (40–50) of monomers and unfolds two β-hairpins from each monomer to build the β-barrel, in contrast to the single hairpin from both the aerolysin- and hemolysin-like families [50]–[52]. Why *C. perfringens* synthesizes so many and such a diverse range of β-PFTs is intriguing. Have these β-PFTs evolved from a common clostridial ancestor gene or have they been acquired by horizontal gene transfer from other bacteria and subsequently evolved in *C. perfringens*? A basic role of bacterial β-PFTs probably concerns the uptake of nutrients from eukaryotic cells. But the benefits for *C. perfringens* of these different β-PFTs is not evident. It might be hypothesized that they contribute to *C. perfringens* adaptation to specific hosts or ecological niches.

In summary, NetB, Delta toxin and Beta toxin are three toxins secreted by *C. perfringens* that are both related to the *S. aureus* leukocidins and, in the case of NetB and Beta toxin, are important pathogenic factors in industrial livestock diseases that are emerging following the removal of antibiotics from foodstuffs [53]. Here, we have presented the 2.4 Å X-ray crystallographic structure of *C. perfringens* Delta toxin monomeric structure together with a model of the heptameric pore-form which is supported by negative stain electron microscopy images. The structure shows that while there are similarities in the mechanism of membrane recognition between the *C. perfringens* toxins and the *S. aureus* leukocidins, there are also a number of key differences that may explain the altered specificity of these toxins. Such differences will be important in the design of novel therapeutics and/or vaccines.

## Supporting Information

Figure S1 **Multiple sequence alignment of** ***C. perfringens*** **Delta toxin.** (Uniprot ID: B8QGZ7), *C. perfringens* Beta toxin (Uniprot ID: Q9L403), *C. perfringens* NetB (Uniprot ID: A8ULG6), *S. aureus* αHL (aHL; Uniprot ID: P09616; PDB ID: 7AHL), the F component of *S. aureus* leucocidin (LukF; Uniprot ID: P0A077; PDB ID: 1LKF), the F component of *S. aureus* Panton-Valentine leucocidin (LukF-PV; Uniprot ID: O50604; PDB ID: 1PVL), the S component of *S. aureus* Panton-Valentine leucocidin (LukS-PV; Uniprot ID: Q783R1; PDB ID: 1T5R), the F component of *S. aureus* γ-Hemolysin (LukF-gHL; Uniprot ID: Q931F3; PDB ID: 3B07) and the S component of *S. aureus* γ-Hemolysin (Hlg2-gHL; Uniprot ID: P0A071; PDB ID: 3B07). Secondary structures elements (arrows for β-strands and coils for α- or 3_10_ helices) are shown in red and at the top for Delta toxin and at the bottom for αHL. Secondary structures elements for the leucocidins are boxed. The predicted Stem domain is in yellow. Sequence identity and homology are in red and grey, respectively. The Figure has been made using ESPript program [54].
(TIFF)
