# Supplementary figures and images for: Structural Insights into Clostridium perfringens Delta Toxin Pore Formation
Source: PLoS One. 2013 Jun 21;8(6):e66673. doi: 10.1371/journal.pone.0066673 (PMC3689675; doi:10.1371/journal.pone.0066673)

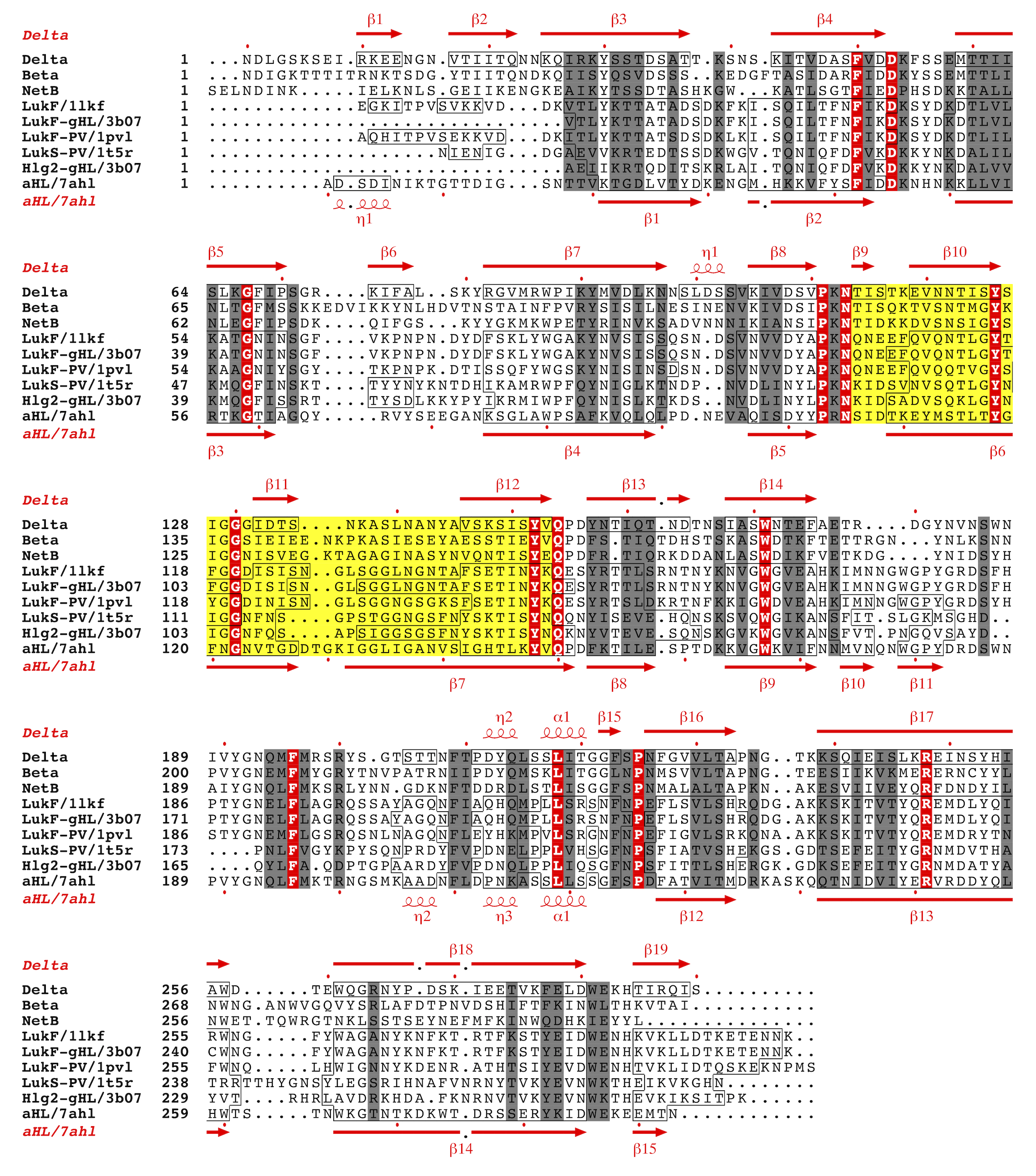

Supplement: Figure S1 — Multiple sequence alignment of C. perfringens Delta toxin. (Uniprot ID: B8QGZ7), C. perfringens Beta toxin (Uniprot ID: Q9L403), C. perfringens NetB (Uniprot ID: A8ULG6), S. aureus αHL (aHL; Uniprot ID: P09616; PDB ID: 7AHL), the F component of S. aureus leucocidin (LukF; Uniprot ID: P0A077; PDB ID: 1LKF), the F component of S. aureus Panton-Valentine leucocidin (LukF-PV; Uniprot ID: O50604; PDB ID: 1PVL), the S component of S. aureus Panton-Valentine leucocidin (LukS-PV; Uniprot ID: Q783R1; PDB ID: 1T5R), the F component of S. aureus γ-Hemolysin (LukF-gHL; Uniprot ID: Q931F3; PDB ID: 3B07) and the S component of S. aureus γ-Hemolysin (Hlg2-gHL; Uniprot ID: P0A071; PDB ID: 3B07). Secondary structures elements (arrows for β-strands and coils for α- or 310 helices) are shown in red and at the top for Delta toxin and at the bottom for αHL. Secondary structures elements for the leucocidins are boxed. The predicted Stem domain is in yellow. Sequence identity and homology are in red and grey, respectively. The Figure has been made using ESPript program [54]. (TIFF) [file pone.0066673.s001.tiff]
